# Antibacterial and cytotoxic activity assessment of *Channa striatus* (Haruan) extract

**DOI:** 10.14202/vetworld.2020.508-514

**Published:** 2020-03-19

**Authors:** Nur Zulaikha Mat Zawawi, Rumaizi Shaari, Muhammad Luqman Nordin, Ruhil Hayati Hamdan, Tan Li Peng, C. W. Salma C. W. Zalati

**Affiliations:** 1 Department of Clinical Studies, Faculty of Veterinary Medicine, Universiti Malaysia Kelantan, Locked Bag 36, Pengkalan Chepa, 16100 Kota Bharu, Kelantan, Malaysia; 2 Department of Preclinical Studies, Faculty of Veterinary Medicine, Universiti Malaysia Kelantan, Locked Bag 36, Pengkalan Chepa, 16100 Kota Bharu, Kelantan, Malaysia; 3 Department of Paraclinical Studies, Faculty of Veterinary Medicine, Universiti Malaysia Kelantan, Locked Bag 36, Pengkalan Chepa, 16100 Kota Bharu, Kelantan, Malaysia

**Keywords:** antibacterial properties, *Channa striatus*, feline periodontitis, periodopathogen

## Abstract

**Background and Aim:**

*Channa striatus* extract, a freshwater snakehead fish known as Haruan, is popular in Southeast Asia for consumption and as a traditional therapeutic remedy for wound healing. *C. striatus* is also used in osteoarthritic for its anti-inflammatory. The aim of this study was to determine the presence of antibacterial properties of *C. striatus* extract against oral bacteria and to investigate the cytotoxic activity against Vero cells.

**Materials and Methods:**

The authors prepared *C. striatus* extract in chloroform-methanol solvents. Next, the authors took subgingival microbiological samples from 16 cats that had periodontal disease. The authors determined the antibacterial properties of *C. striatus* extract against the isolated bacteria using the disk diffusion method and a broth microdilution-based resazurin microtiter assay. Finally, the authors used the Vero cell line to evaluate the cytotoxic activity, and they assessed the cell availability using the 3-(4,5-dimethylthiazol-2-yl)-2,5-diphenyltetrazolium bromide (MTT) assay.

**Results:**

The results showed weak antibacterial activity of *C. striatus* extract against *Pseudomonas* spp. and *Escherichia coli*. In addition, the authors found that minimum inhibition concentration values ranged between 400 and 500 mg/mL, and minimum bactericidal concentration values ranged between 650 and 550 mg/mL. However, the cytotoxic results were promising, showing that *C. striatus* extract increased the cell viability and growth when it was at a higher concentration. The extract also promotes growth and cell proliferation.

**Conclusion:**

These findings suggest that *C. striatus* extract promoted cell proliferation *in vitro* and could be a plausible therapeutic wound healing alternative for periodontal disease in cats.

## Introduction

Periodontal disease is a common problem in feline dentistry that is caused by polymicrobial, multifactorial diseases, but there are many other factors that play a role in the infection for the host [1,2]. Certain bacteria encountered in subgingival plaque are responsible for the formation of periodontal disease, and the absence of appropriate oral hygiene measures causes the build-up of many different types of bacteria along the gum line that may lead to irritation and inflammation [2,3]. The most significant and most predominant anaerobic Gram-negative bacteria in the subgingival area are *Prevotella intermedia*, *Actinobacillus actinomycetemcomitans*, and *Porphyromonas gingivalis* [4]. The aerobic bacteria that the authors identified in the previous study were *Pasteurella multocida*, *Streptococcus* spp., *Enterococcus* spp., *Staphylococcus* spp., *Bacillus cereus*, *Escherichia coli*, and *Pseudomonas aeruginosa* [5].

At present, there is increasing bacterial resistance against routine antibiotics for the treatment of microbial disease [6,7]. Hence, scientists seek an alternative agent for allopathy, such as plant- and animal-based resources [8]. One promising candidate is *Channa striatus* extract. *C. striatus*, known locally in Malaysia as Haruan or snakehead fish, belongs to the Channidae family and is widely distributed in Southeast Asian countries [9]. *C. striatus* is well known for wound healing in postpartum cases in human traditional medicine [10]. At present, Malaysian popularly uses the extract for wound healing, anti-nociceptive, and anti-inflammation properties, inducing stem cell proliferation, stimulating platelet aggregation, and enhancing cognitive functioning [9,11,12]. The extract contains arginine and glutamine, which play important roles in wound repair and antibacterial activity [13].

Bacterial resistance is spreading throughout the world, primarily due to uncontrolled use of antibiotics [14]. As stated in several studies, *C. striatus* extract has potential antimicrobial, antifungal, and ­anti-nociceptive properties [15]. Therefore, researchers should investigate the properties of *C. striatus* extract in feline periodontal disease, focusing specifically on its antimicrobial aspects. In addition, there was no empirical investigation into the antimicrobial action of *C. striatus* on the feline oral microorganism. In this study, the authors evaluated that the antimicrobial and cytotoxic activity of *C. striatus* fillet extract was *in vitro*.

Therefore, the aim of this study was to investigate the antimicrobial activity against the feline oral microorganism and the cytotoxic activity of *C. striatus* extract.

## Materials and Methods

### Ethical approval

The study was approved by Institutional Animal Care and Use Committee (IACUC), Faculty of Veterinary Medicine, University Malaysia Kelantan (UMK/FPV/ACUC/PG/2018/1).

### Preparation of C. striatus extract

**Figure-1:**
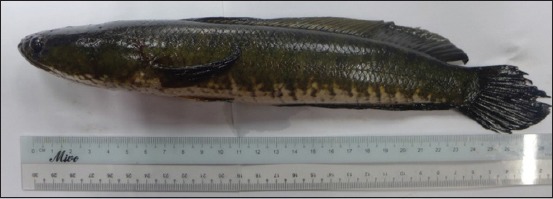
Adult Channa striatus known as Haruan.

The authors purchased *C. striatus* (Bloch. 1793) from the wet market in Rural Transformation Centre, Kota Bharu, Kelantan. The appearance of *C. striatus* is shown in Figure 1. Next, the authors prepared *C. striatus* extract according to the method described by Zakaria *et al*. [16]. The authors prepared the aqueous supernatant of *C. striatus* using a 2:1 chloroform: methanol (CM) solvent. Then, the authors directly mixed the fillet with CM at a ratio of 1:2 (w/v). The authors left the mixtures overnight and then filtered them. Then, the authors left the supernatant for 30 min to settle it down to two layers. The authors collected the upper layer, which was an aqueous supernatant of Haruan (ASH) layer, and removed any methanol residue through evaporation. The authors freeze-dried the pure aqueous solution for 48 h to completely remove the water portion of aqueous extract of *C. striatus*. Finally, the authors obtained and weighed the dried sample. They calculated the yield percentages using the following formula [17]:


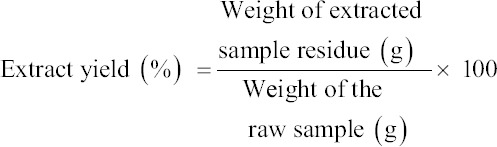


### Bacterial isolation and identification of feline periodopathogen

The authors took the subgingival microbiological samples from 16 cats that had periodontal disease. Next, the authors sampled bacteria using sterile absorbent endodontic paper points (Sure-endo, Korea). The authors inserted the paper points and left them in the dental pocket for 10 s. The authors immediately stored the paper points in thioglycolate broth (Merck, Germany) and nutrient broth (Oxoid, United Kingdom) as transport media.

The authors cultured the samples on blood agar and MacConkey agar (Oxoid, United Kingdom) in aerobic condition. Next, the authors incubated the plates at 37°C for 24 h. The authors further identified isolates using Gram staining, biochemical test (citrate test, triple sugar iron agar, urease, sulfide, indole, motility, methyl red, Voges–Proskauer, and Analytical Profile Index 20E kits [bioMerieux, France]). Finally, the authors further tested the most isolated microorganisms for antimicrobial activity by screening them with *C. striatus* extract.

### Disk diffusion method

The authors performed the antibacterial activity by the disk diffusion method according to the Clinical Laboratory Standards Institute method [18-20]. The authors adjusted the turbidity of bacteria to 1.5×10^6^ CFU/mL in physiological saline. Next, the authors inoculated the prepared inoculum onto Mueller-Hinton agar (Oxoid, United Kingdom) plates. The authors then impregnated the blank disks with 20 µL extract stock solution (1000 mg/mL). The authors dried all impregnated disks overnight in the oven at 40°C. The authors then carefully placed the impregnated dry disks on the agar plates at equidistance points using sterile forceps. Each plate consisted of antibiotic discs and three other disks impregnated with extracts. Then, the authors freshly prepared two negative control disks in 1:2 methanol and ethanol solvent and sterile distilled water before leaving them to air-dry. The authors used enrofloxacin (5 µg) disk (Oxoid, United Kingdom) as a positive control [21]. After standing for 30 min, the authors incubated the plates in an inverted position at 37°C for 24 h. Finally, the authors determined antimicrobial activity by measuring the size of the inhibition zone to the nearest mm, and they recorded the results.

### Broth microdilution-based resazurin microtiter assay

The preparation of inoculum was done according to Aminah *et al*. [17]. The concentration of the bacterial inoculum was 5×10^5^ CFU/mL. Next, the authors completed the Resazurin Microtiter Assay method (REMA) according to Mota *et al*. [22]. The authors performed the REMA microdilution method in sterile flat-bottom 96-well microplates. The authors sterilized all solutions using a 0.22 µm membrane filter. Briefly, the authors added 10 µL of overnight inoculum (5×10^5^ CFU/mL) into each well containing 100 µL of different concentrations of the extracts (50 mg/mL-800 mg/mL) with Mueller-Hinton Broth 2 (Sigma, India).

Next, the author incubated the solutions in incubator shaker (Heidolph UNIMAX 1010, European) at 180 rpm, 37°C for 24 h. The authors used enrofloxacin (1000 µg/mL) as a control drug for each set of microplate [23]. The authors added resazurin to all wells and further incubated for 2-4 h at 37°C for the observation of color changes. The authors observed growth inhibition by visual inspection of the turbidity and color changes of the mixture. The authors determined the minimum bactericidal concentration when there was no colony growth from the directly plated contents of the wells. The authors serially diluted the content of the wells that showed the indication of growth inhibition to quantify an endpoint killing of the bacteria. The authors also prepared positive and negative cultures [24]. Finally, the authors carried out the evaluation of minimum inhibitory concentration (MIC) in triplicates.

### Cell preparation and maintenance

The authors grew African green monkey kidney (Vero) cells in Dulbecco’s modification of Eagle’s medium (DMEM), supplemented with 10% heat-inactivated fetal bovine serum (Nacalai Tesque), 1% antibiotic-antimycotic (10,000 units/mL of penicillin, 10,000 µg/mL of streptomycin, and 25 µg/mL amphotericin B) as a complete growth medium (CGM). The authors thawed the cells slowly from the liquid nitrogen to −80°C and then placed in 36.5°C water bath before culture. The authors transferred 1 mL of cells to 15 mL and centrifuged them at 1200 rpm for 5 min. Then, the authors removed the supernatant and resuspended the pellets with 1 mL of CGM. The authors then transferred the 1 mL of cell suspension to 75 cm^2^ cell culture flask. Next, the authors added 10 mL of complete growth media to the flask and incubated it at 37°C with 5% CO_2_ in incubator. The authors subcultured the cultured flask into another flask once it reached 80% confluency. The cells were detached with 1.5 mL of 0.25% trypsin-EDTA after the removal of old media, and they were washed with 5 mL phosphate-buffered saline. Finally, the authors checked the cells microscopically daily to ensure the cells were in healthy condition.

### Cytotoxic assay

Briefly, the authors prepared about 1 mg/mL extract, and they centrifuged the extract. The authors extracted the supernatant layer and sterilized it using a syringe filter. The authors serially diluted the sterile extract to the desired concentration with 10% DMEM. The authors assessed that the cytotoxicity of the extracts (dissolved in distilled water) against African green monkey kidney cell line (Vero) was assessed by the microculture tetrazolium test (MTT) reduction assay (Nacalai Tesque, Japan) according to the method described by Nordin *et al*. [25] and Dzoyem *et al*. [26]. The authors seeded about 100 µL of Vero cell into 96-well microtiter plates at a density of 1×10^5^ cells/mL, and they incubated it at 37°C and 5% CO_2_ for 24 h to allow cell attachment.

Then, the authors discarded the media, and they added 10 µL of different concentrations of extract and 90 µL of 2% DMEM into each well. Each concentration of treated cells, untreated cells, and the blank was performed in triplicates. After a 72 h incubation of the treatment period, the authors discarded 90 µL of media and added 10 µL of MTT solution into each well. The authors incubated the microtiter plates for 4 h. Then, the authors added 100 µL of solubilization solution into the wells. Following this step, the authors pipetted solution in each well to dissolve the form alone. The authors determined cell viability by measuring absorbance at 570 nm using a microplate reading spectrophotometer (Molecular Devices Co., USA). The authors calculated the percentage of cell viability (%) based on the following formula [26]:


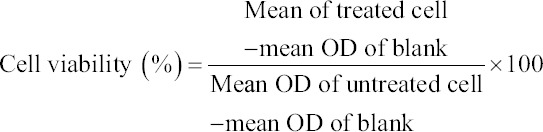


### Statistical analysis

The authors expressed the result values of each experiment as mean (n=3) ±standard deviation. The authors performed statistical analysis with GraphPad Prism version7.0 (www.graphpad.com) to compare treated cells and untreated cells using one-way ANOVA. A significant difference was indicated by p<0.05.

## Results

### *C. striatus* extract yield

From each 3.2 kg of the wet weight of *C. striatus* that was used, approximately 29.5 g of light brown CM extract was obtained. The authors then prepared this extract was in the desired doses used for the antibacterial and cytotoxic assay. The extraction yield obtained in this experiment was 0.92%.

### Bacterial isolation and identification

The most isolated periodopathogen were *Staphylococcus* spp., *Pseudomonas* spp., *Klebsiella* spp., *Streptococcus* spp., *P. multocida*, and *E. coli*. In addition, the authors tested these microorganisms for antimicrobial activity screening with *C. striatus* extract.

### Antibacterial sensitivity test (disk diffusion method)

The values were the mean of three experiments. The values given are the diameter of zone of inhibition (mm) including a disk diameter of 5.5 mm. Antibacterial activity of *C. striatus* extract (800 mg/mL) showed that *Pseudomonas* spp. was inhibited with a zone diameter 7.3±0.6 mm by disk diffusion method.

As shown in Table-1, *C. striatus* extract exhibits different antimicrobial activities depending on which oral bacteria are tested. *C. striatus* extract exhibited a weaker antimicrobial activity against *Pseudomonas* spp. and *E. coli* (MIC range: 400-500 mg/mL; MBC range: 650-550 mg/mL). The MIC test showed that the extract with a concentration of 500 mg/mL was able to inhibit the growth of *E. coli* and *Pseudomonas* spp.

**Table-1:** Antibacterial activity of *Channa striatus* extract.

Feline oral microorganisms	Antibacterial activity; MIC/MBC (mg/mL)
*Staphylococcus* spp.	NT
*Pseudomonas* spp.	400/650
*Klebsiella* spp.	NT
*Streptococcus* spp.	NT
*Escherichia coli*	500/550

MIC=Minimum inhibitory concentration, MBC=Minimum bactericidal concentration

### Cytotoxicity test

**Figure-2:**
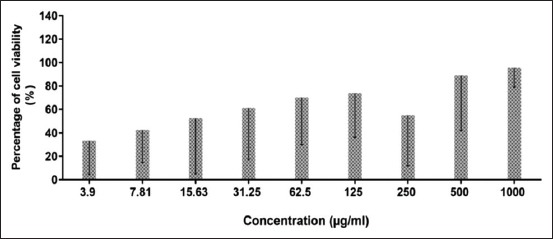
Viability (%) of cells treated with Channa striatus extract at different doses by MTT assay after 72 h. All values are expressed as mean ± standard deviation (n=3).

Figure-2 shows the viability of the cells treated with *C. striatus* extract. In general, the cell proliferates in a dose-dependent manner. Based on research by the National Cancer Institute (United States), there are four group classifications for cytotoxicity evaluation: Very active (IC_50_ ≤20 µg/mL), moderately active (IC_50_ >20-100 µg/mL), weakly active (IC_50_ >100-1000 µg/mL), and inactive (IC_50_ >1000 µg/mL) [27]. The cell viability in this study showed proliferation of the cell, and no IC_50_ was identified in this study after 72 h of extract exposure. However, in this study, the higher concentrations of extract (15.63, 31.25, 62.5, 125, 500, and 1000 µg/mL) did not affect the viability of the cell. Interestingly, the higher concentration of *C. striatus* extract promotes Vero cell growth. However, statistically, there was no significant difference between the cellular viability values at 72 h (p> 0.05) (Table-2).

**Table-2:** Percentage (%) of cell viability of *Channa striatus* extract on Vero cell line after 72 h of treatment.

*Channa striatus* extract concentration (µg/mL)	3.9	7.81	15.63	31.25	62.5	125	250	500	1000
Percentage of cell viability	40.20 ±26.1	45.38 ±19.8	66.47 ±42.5	67.17 ±35.6	73.35 ±26.5	76.08 ±23.2	62.53 ±37.3	91.61 ±24.9	95.75 ±7.20

Values are expressed as mean±standard deviation (n=3) per plate for 3 times experiments. The comparison between treated cells and untreated cells (control) was evaluated using one-way ANOVA. *p<0.05 denotes significant difference as compared to untreated cell (control)

## Discussion

Periodontal disease is one of the most common diseases in cats that cause by multiple factors, especially oral bacteria. Thus, it is crucial to investigate the antimicrobial and cytotoxic effects of the extract. This study will enhance researchers’ understanding of the natural, therapeutic, and healing effects of *C. striatus* extract on the gingiva. *C. striatus* also has the potential to be an alternative option to replace corticosteroid therapy in feline periodontitis.

In this study, the authors selected five bacteria commonly isolated in feline periodontal disease. These bacteria have indirectly contributed to pathogenesis and biofilm formation in cases of periodontal disease. *C. striatus* extract shows antibacterial effects against *E. coli* and *Pseudomonas* spp. because both are Gram-negative bacteria that have hydrophilic characteristics. Gram-negative bacteria have hydrophilicity higher than Gram-positive bacteria, which allow the permeability of hydrophobic agents in the cell wall [28,29].

Gram-negative bacteria have thinner cell walls compared to Gram-positive bacteria. Thus, researchers believe that the hydrophobic charge of the extract increases the amount of extract that is absorbed into the cell wall of Gram-negative bacteria. In addition, the previous study stated that the permeability of the cell walls of bacteria depends on the charge, hydrophobicity, and amphipathic properties of the extract [28]. Based on these hydrophilic, thin cell wall, and extract characteristics, the authors believe that it will contribute to permeability of the extract and to the antibacterial nature of the extract in inhibiting bacteria growth. However, further research should investigate the action of *C. striatus* extract in its antibacterial characteristics.

The previous studies regarding the antibacterial properties of *C. striatus* extract demonstrated that the mucus extract had adequate antimicrobial properties against a few strains of bacteria, including *P. aeruginosa*, *E. coli*, and *Staphylococcus aureu*s [30,31]. This finding was also supported by Kumar *et al*. [32]. In addition, in Kumar *et al*. study, the blood and gills of *C. striatus* extract also inhibited *E. coli* and *Salmonella* enteritidis, but the commercialized antibiotic chloramphenicol (positive control) showed resistance toward the tested *E. coli*.

In this study, the MIC result showed that a concentration of 500 mg/mL was able to inhibit *E. coli* and 400 mg/mL was able to inhibit *Pseudomonas* spp. This suggests that the higher concentration is needed to have an antibacterial effect. Thus, these results indicate that *C. striatus* extract exhibits weak antimicrobial activity against *E. coli* and *Pseudomonas* spp. Another study used CM extract from fillet to investigate the antifungal, antibacterial, and anti-nociceptive properties [33]. The authors of this study found that there is no anti-staphylococcal activity in *C. striatus* fillet extract. Based on these findings and the previous study, mucus extract exhibits good antibacterial properties compared with fillet extract [34].

Compared with the data in minimum inhibition concentration, data from the disk diffusion method (Table-1) showed a contradiction in the antibacterial activity. The disk diffusion for the detection of antibacterial activity for the herbal drug depended on the hydrophobic and polarity characteristic of the extract solvent used. These findings, which were supported by Moreno *et al*.[35] show that the absence of inhibition zones is not indicative of inactive antibacterial extract. Rather, it is due to low polarity of the solvent that caused the authors to find no inhibition [35,36]. In this study, there was no uniform diffusion through the agar media due to hydrophobicity of natural extract to cause the absence of inhibition effect.

The researcher could consider *C. striatus* extract a weak antimicrobial agent. This finding is contrary to the authors’ expectations, and a previous study by Dhanaraj *et al*. [30] did not support that *C. striatus* extract has a high potential for antimicrobial properties. Researchers believe that a possible factor that plays a role in these results is the part of *C. striatus* that was used in this study and its compound. The main compounds detected in this *C. striatus* fillet extract were amino acids (glycine, glutamic acid, arginine, and aspartic acid) and fatty acids (eicosapentaenoic acid, docosahexaenoic acid, palmitic acid, oleic acid, stearic acid, and arachidonic acid), which are important in influencing pain-sensing and wound healing [37-39].

However, none of the scientific literature reported the antimicrobial content in *C. striatus*, such as terpenoids or α-pinene. This leads the authors to believe that *C. striatus* fillet extract might have weak antimicrobial activity due to the lack of an antimicrobial compound. Thus, the selection of *C. striatus* extract parts is important because there is a different bioactive compound in each part. The interesting about *C. striatus* fillet extract is that it has notable wound healing properties [12]. Therefore, this study provides important information on the investigation of the antibacterial activity of fillet extract and the safe usage of *C. striatus* on normal cells.

In the Vero monkey kidney cell, *C. striatus* extract shows a higher concentration of extract, the higher the cell availability. Interestingly, this shows that *C. striatus* could promote cell growth and is safe to use in high concentrations. This result is consistent with the previous studies, in which the extract promotes cell proliferation of mesenchymal stem cell and fibroblast cells (3T3 cells) [12,13]. Based on this finding, the authors believe that the ability to promote wound healing also related to an amino acid compound of the extract that promotes cell growth. Thus, the authors believe that the application of *C. striatus* extracts in higher concentrations will enhance the wound healing process and will help bacteriostatic of the oral bacteria (Table-1).

The application of *C. striatus* in wound healing is quite common. Most of the previous studies have successfully shown a wound-healing effect after the use or consumption of *C. striatus* in post-operative patients with cesarean cases [10]. The finding of weak antibacterial in this study is crucially important because the optimum healing effect happens when the extract is applied to a clean wound. However, in contaminated wound cases, normal wound dressing using antibacterial solution and antibiotics must also be given by clinicians.

## Conclusion

Antibiotic-resistant bacteria and steroidal side effects during prolong treatment continue to arise and become the concern in global public health threat, nowadays. Therefore, scientific efforts have been made to study and develop new compounds based on natural sources to be used beyond current conventional antibiotic drugs and steroidal treatments. To the best of the authors’ knowledge, the present work is the first study of *C. striatus* fillet extract on feline oral microorganisms. This extract has antibacterial effects against *E. coli* and *Pseudomonas* spp. due to its cellular permeability. An interesting finding is ability of this extract to promote cell growth and the potential for *C. striatus* extract to be considered as a candidate for therapeutic treatment in future for wound healing, especially in feline gingivitis.

## Authors’ Contributions

NZMZ designed and performed the study and wrote the manuscript. CWSCWZ, MLN, RHH, TLP, and RS managed the analyses of the study and were involved in data analysis. RS directed and supervised the project. All authors read and approved the final manuscript.
